# Radiation induced glioma in a sexagenarian: A case report

**DOI:** 10.1097/MD.0000000000025373

**Published:** 2021-04-23

**Authors:** You-Heng Peng, Seidu A. Richard, Zhigang Lan, Yuekang Zhang

**Affiliations:** a Department of Neurosurgery, West China Hospital, Sichuan University; 37 Guo Xue Xiang Road, Chengdu, Sichuan, P. R. China; b Department of Medicine, Princefield University, P. O. Box MA 128, Ho-Volta Region, Ghana, West Africa.

**Keywords:** chemotherapy, medulloblastoma, radiation, radiotherapy, sexagenarian, surgery

## Abstract

**Introduction:**

Radiation induced gliomas often occurs after radiation therapy for other brain tumors. Medulloblastoma often occurs in children and its associated radiation-induced glioblastoma multiforme's (GBM) after radiotherapy often has a long latency period. Our case is very unique because the medulloblastoma was detected at an advance age and the latency period of radiation-induced GBM was relatively shorter.

**Patients Concerns:**

A 64-year-old male was first admitted at our hospital in March 2018 with dizziness, vomiting, and blurred vision.

**Diagnosis:**

Magnetic resonance imaging of brain revealed a lesion with local mixed density and mass enhancement in left cerebellar region. Histopathology established medulloblastoma (World Health Organization) grade 4 and a classic histological subtype after surgery.

**Intervention:**

Surgical resection followed by radiation therapy were the initial therapeutic modalities.

**Outcomes:**

In April 2019, the patient was readmitted with dizziness and blurred vision. Magnetic resonance imaging showed the left cerebellar hemisphere bulky enhancement lesion. Again, a multimodal therapy comprising surgical resection, radiation therapy as well as chemotherapy was adapted after histopathology established GBM.

**Lesion:**

Radiotherapy for medulloblastoma patients at advance ages is a critical predisposing factor for the development of radiation-induced GBM in a very short period of time. We suggest that, radiotherapy as adjuvant therapy for medulloblastoma patients at advance ages should be chosen with extreme caution.

## 1 Introduction

Radiation induced gliomas often occurs after radiation therapy for other brain tumors.^[1–5]^ These transformational lesions are important complication of radiotherapy with incidence rate of about 1% and mainly glioblastoma multiforme's (GBMs).^[1]^ Tumors such as medulloblastoma, genital tumor and Burkitt lymphoma have transformed into GBM after radiotherapy^[3,6,7]^ Since Kleriga et al reported the first case of radiation-induced GBM developing from medulloblastoma after radiation therapy in 1978, there have been dozens of similar cases reported over 40 years period.^[2]^

Radiation-induced genetic changes have been implicated as the cardinal causes of radiation-induced GBMs.^[6]^ Nevertheless, the processes involved in radiation-induced mutations resulting in oncogenes and tumor facilitation are not clear.^[6]^ Previous reports have shown that, GBM induced by radiation often resulted in poor prognosis because of the highly invasive nature of the lesions.^[7]^ Medulloblastoma often occurs in children and its associated radiation-induced GBM after radiotherapy often has a long latency period.^[3]^

Unlike most of the previous reported cases, we report a case of a male sexagenarian with radiation-induced GBM who received radiotherapy for medulloblastoma 10 months prior. Thus, our case was very unique because the medulloblastoma was detected at an advance age and the latency period of radiation-induced GBM was relatively short.

## 2 Case report

A 64-year-old male was first admitted at our hospital in March 2018 with dizziness, vomiting, and blurred vision. Neither the patient nor his family had any known genetic disease predisposing to cancer. Neurological examination was unremarkable. Inferior cranial nerve examination did not yield much. General physical examination was unremarkable. Routine laboratory investigations were grossly normal. Chest X-ray and electrocardiogram were essentially normal.

**Figure 1:**
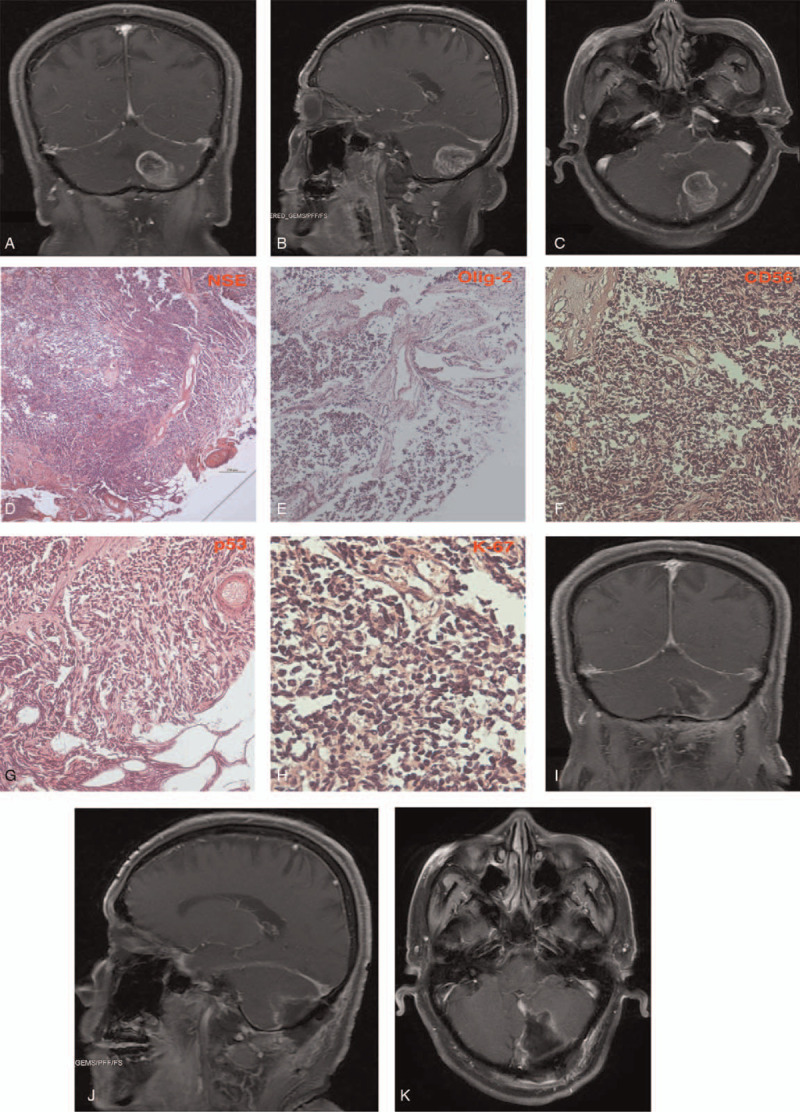
A-C, are preoperative MRIs showing the lesion with local mixed density and mass enhancement in left cerebellar region. A- coronal, B-sagittal, C- axial. D-H, are histopathology imaging confirming the diagnosis of medulloblastoma (WHO) grade 4 and a classic histological subtype. D- Neuron specific enolase (NSE), E- Oligodendrocyte transcription factor (Olig-2), F- CD56, G-p53 and H-30-40% K-67. I-K, are postoperative MRIs showing total resection of the medulloblastoma. I- coronal, J-sagittal, K- axial.

Magnetic resonance imaging (MRI) of brain revealed a lesion with local mixed density and mass enhancement in left cerebellar region (Fig. 1 A-C). The lesion measured 2.8 x 3.7 x 3.5 cm in diameter. The lesion exerted compressive effect on midline structure displacing them to the right side. The lesion had well defined borders with marked midline oedema in between both cerebellums. Initial diagnosis of a left cerebellar hemisphere malignant tumor was made. The patient was prepared for surgery the next day.

After general anesthesia, the patient was put in the park-bench position with the head fixed in the Mayfield three keys. The suboccipital approach was used to access the tumor. Intraoperatively, we observed a solid lesion with a small amount of purplish red clot and oedema. The lesion was soft and purple with rich blood supply. The borders between the lesion and the surrounding brain tissues were not very clear. The lesion was completely resected. After securing total hemostasis, the bone flap was replaced and the muscles and the skin closed in layers.

Histopathological evaluation of the samples revealed neuron specific enolase, Oligodendrocyte transcription factor, CD56, p53 as well as 30% to 40% K-67 positive (Fig. 1 D-H) which was consistent with the diagnosis of medulloblastoma (World Health Organization) grade 4 and a classic histological subtype. Postoperative MRI showed total resection of the lesion (Fig. 1, I-K). The patient's symptoms improved significantly, and he was discharged from hospital after scheduling him for radiotherapy.

In May 2018 the patients received radiotherapy. Intraoperatively, the tumor bed was infiltrated with 5400cGy/180cGy/13 times, while the whole brain and spinal cord was infiltrated with 3060cGy/180cGy/17 times. Post-operative cause was uneventful and the patient was sent home after 3 days observation in the ward. Routine scheduled visits every six months were arranged.

**Figure 2:**
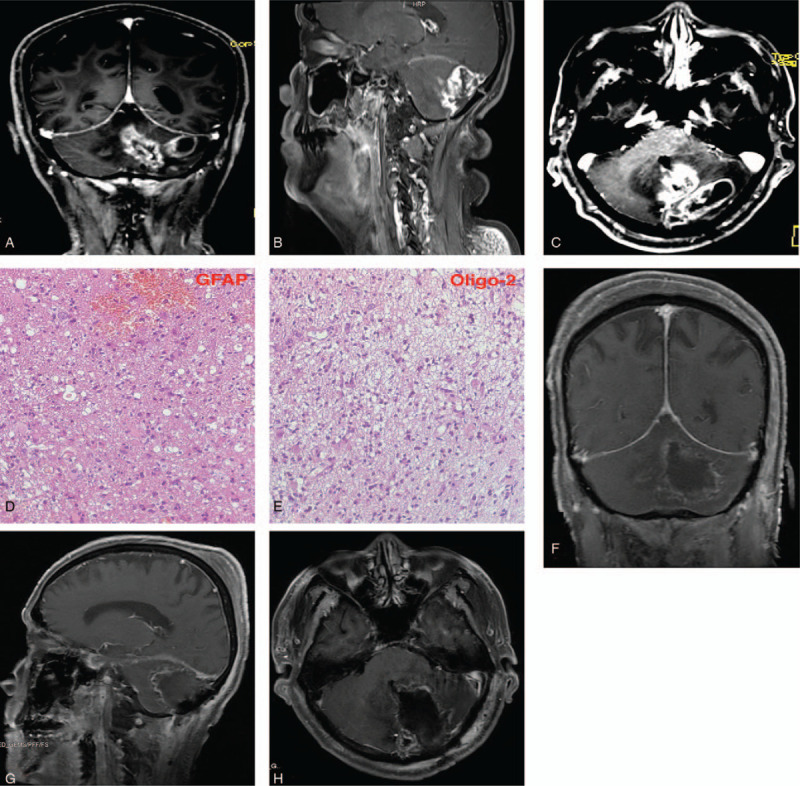
A-C, are MRIs showing a recurrent left cerebellar hemisphere bully enhancement lesion with more enhanced intensity compared with the post-operative MRI. A- coronal, B-sagittal, C- axial. D&E, are immunohistochemically images confirming the diagnosis of radiation-induced glioblastoma multiforme (GBM). D-glial fibrillary acidic protein (GFAP) and E- Oligodendrocyte transcription factor (Olig-2). F-H, are Post-operative MRIs showing total resection of second the lesion. F- coronal, G-sagittal, H- axial.

In April 2019, the patient was readmitted with dizziness and blurred vision. Examination of the inferior cranial nerves did not yield much. General physical examination was unremarkable. routine laboratory investigations were grossly normal. Chest X-ray and electrocardiogram were essentially normal. MRI showed a recurrent left cerebellar hemisphere bulky enhancement lesion (Fig. 2, A-C) in the field of the radiation therapy with more enhanced intensity compared with the postoperative MRI. The lesion measured 2.5 x 2.7 x 2.3 cm in diameter. The lesion had well defined borders. Based on his previous history above, a diagnosis of a recurrent medulloblastoma was made. The patient was prepared for surgery the next day.

After general anesthesia, the patient was put in the park-bench position with the head fixed in the Mayfield three keys again. The suboccipital approach via the old incisional scar was used to access the tumor again. Intraoperatively, we observed a solid lesion with rich blood supply. The borders between the lesion and the surrounding brain tissues were not very clear. The lesion was again resected. After securing total hemostasis, the bone flap was replaced and the muscles and the skin closed in layers.

Initial examination of the tumor samples revealed massive hemorrhage and necrosis, with poor tumor differentiation. Immunohistochemically, the tumor cells were positive for glial fibrillary acidic protein and Oligo-2 (Fig. 2, D and E). However, the cells were negative for synaptophysin, p53 and Epithelial membrane antigen. In addition, no positive results were found in isocitrate dehydrogenase 1 and other gene mutation analysis, except that C to T missense codon at 228 was detected in telomerase reverse transcriptase promoter mutation analysis. The histological morphology, immunohistochemical analysis and molecular detection above are consistent with GBM.

Post-operative MRI showed total resection of the second lesion (Fig. 2, F-H). The patient's symptoms improved significantly, and he was discharged from hospital. He was further treated with gamma knife radiotherapy (90Gy and 60Gy in 1.8 Gy fractions) and oral Temozolomide 150 mg/m^2^ for 5 days; repeat at 28-day cycles and maintenance dose of 200 mg/m^2^ for 5 days/28-day cycle. One and half year's follow-up revealed no recurrence of the lesion. Nevertheless, we are still following the patient closely.

## 3 Discussion

Radiation-induced intracranial tumors have been observed within the brain, meninges, bones, as well as the connective tissue elements of the CNS.^[3,8,9]^ However, meningiomas and gliomas are the most frequently observed secondary tumors.^[3,8,9]^ In 1978, Kleriga et al reported the first case of radiation-induced GBM caused by medulloblastoma after central nervous system radiotherapy.^[2]^ Over a 42 years period, many literatures have reported cases of medulloblastoma transforming into GBM after radiotherapy. The window periods of transformation in all the cases reported in literature was within 15 years.^[3,7]^

Medulloblastoma mainly occurs in children under 15 years old. Nevertheless, the incidence of medulloblastoma in adult is about 0.4% to 1%, and most of them are under 40 years old.^[10,11]^ Thus, the detection of medulloblastoma in the elderly over 60 years old is extremely rare. We report the first case of radiation-induced GBM after treatment of medulloblastoma in a sexagenarian. The classical symptomatology of medulloblastoma often comprise of ataxia, difficulties with handwriting or other motor skills as well as anomalies in vision or strabismus.^[11]^ Our patient presented with dizziness, vomiting as well as blurred vision.

Cranial and spinal MRI with contrast sequences imaging is often used to diagnose as well as stage medulloblastoma.^[11,12]^ Thus, MRI was the key radiological modality used to identity the lesion which composed of local mixed density and mass enhancement in left cerebellar region in our patient. Multimodal therapy comprising surgical resection, radiation therapy as well as chemotherapy have demonstrated to be the standard treatment of medulloblastoma.^[11,13]^ Our patient was treated as per the standard treatment modalities above. Histopathology revealed neuron specific enolase, Oligodendrocyte transcription factor, CD56, p53 as well as 30% to 40% K-67 positive which was coherent with the diagnosis of medulloblastoma (World Health Organization) grade 4 and a classic histological subtype.

Surveillance imaging with MRI, including gadolinium contrast-enhanced images are typically scheduled every 3 months during the first year after therapy and is gradually spaced out over time.^[14]^ Studies have demonstrated that, GBM show typical MRI findings like heterogeneous enhanced bulky mass with central necrosis within several months usually 1.25 to 10 months.^[14–17]^ Furthermore, the average time frame from the initial to final radiological diagnosis of glioblastoma has been 4.5 months.^[14–17]^ We observed a bulky lesion at a site we totally resected a medulloblastoma and further treated the patient with radiotherapy about 10 months after completion of therapy. We initially thought the bulky lesion observed on MRI was a recurrence of the resected medulloblastoma but histopathology established GBM. Thus, the diagnosis of radiation-induced GBM was established.

Per Cahan et al criteria for the diagnosis of radiation-induced tumor^[18]^:

(1) the second tumor must occur in the field of radiation for the treatment of the primary tumor;
(2) the latency between radiotherapy and the discovery of the second tumor must be prolonged, usually several years;
(3) there must be a histological difference between primary and secondary tumors;
(4) the rarity of tumor in patients who have not yet received radiotherapy;
(5) no known genetic or other predisposing conditions for secondary malignancies.
(6) Although the latency period of the second tumor in case was short, it still meets the criteria of radiation-induced GBM as established by Cahan et al. Nevertheless, Cahan et al criteria for the diagnosis of radiation-induced tumor fall short of age variation.^[18]^

Salvati et al. and Wang et al. believed that the age of patients at the first radiotherapy may be an important factor in the development of radiation-induced GBM.^[7,19]^ Our case suggests that, radiotherapy for medulloblastoma patients at advance ages is a critical predisposing factor for the development of radiation-induced GBM in very short period of time contrary to the latency period proposed in Cahan et al criteria. Thus, we suggest that, radiotherapy as adjuvant therapy for medulloblastoma patients at advance ages should be chosen with extreme caution. Studies have shown that, even low doses of radiation were associated with secondary neoplasms in about 10% of patients who developed secondary gliomas after treatment with radiations less than 16 Gy.^[3,18,20]^

Also, multimodal therapy comprising surgical resection, radiation therapy as well as chemotherapy have demonstrated to be the standard treatment of glioblastomas.^[21]^ Our patient was treated in accordance to the standard treatment of glioblastomas. Post-operative radiation is an acceptable treatment option of glioblastomas.^[21]^ As compare to supportive care alone, post-operative radiation often augments survival as well as improved quality of life.^[21,22]^ Based on the above, we further re-treated our patient with radiation therapy although the GBM was caused by radiation. We did not observe recurrence of the GBM after the re-radiation therapy and the quality of the patient improve markedly. Nevertheless, we are still following the patient closely at out-patients department.

Amplification or activating mutations of epidermal growth factor receptor is often observed in primary glioblastomas that arise de novo.^[4,23]^ On the other hand, P53 inactivation as well as amplification of platelet-derived growth factor receptor beta are often observed in secondary glioblastomas usually the low-grade gliomas.^[4,24]^ Furthermore, deletions or mutations of the phosphatase and tensin homolog tumor suppressor gene that results in the up-regulation of protein kinase B oncogenic pathway are often observed in both primary and secondary glioblastomas.^[4]^ Nevertheless, mutations of the phosphatase and tensin homolog tumor suppressor gene are normally deficient in radiation-induced glioblastomas.^[1,4]^ Radiation-induced glioblastomas may also have a lesser proportion of epidermal growth factor receptor secretion as well as a lesser incidence of p16Ink4A alterations than primary glioblastomas.^[1,4]^ In glioblastomas patients, mutations that are observed in methylation of methylguanine methyltransferase, IDH, and telomerase reverse transcriptase have significant prognostic implications.^[21]^

## 4 Conclusion

Radiotherapy for medulloblastoma patients at advance ages is a critical predisposing factor for the development of radiation-induced GBM in a very short period of time contrary to the latency period proposed in Cahan et al criteria. Thus, we suggest that, radiotherapy as adjuvant therapy for medulloblastoma patients at advance ages should be chosen with extreme caution.

## Author contributions

All authors contributed toward data analysis, drafting and critically revising the paper and agree to be accountable for all aspects of the work.

**Conceptualization:** You-Heng Peng, Seidu A Richard, Zhigang Lan, Yuekang Zhang.

**Data curation:** You-Heng Peng, Seidu A Richard, Zhigang Lan, Yuekang Zhang.

**Formal analysis:** You-Heng Peng, Seidu A Richard, Zhigang Lan, Yuekang Zhang.

**Funding acquisition:** Zhigang Lan.

**Methodology:** You-Heng Peng, Seidu A Richard, Zhigang Lan, Yuekang Zhang.

**Resources:** You-Heng Peng, Seidu A Richard, Zhigang Lan, Yuekang Zhang.

**Supervision:** Zhigang Lan, Yuekang Zhang.

**Writing – original draft:** You-Heng Peng, Seidu A Richard.

**Writing – review & editing:** You-Heng Peng, Seidu A Richard, Zhigang Lan, Yuekang Zhang.
